# Laser-Synthesis of NV-Centers-Enriched Nanodiamonds: Effect of Different Nitrogen Sources

**DOI:** 10.3390/mi11060579

**Published:** 2020-06-09

**Authors:** Luca Basso, Mirko Sacco, Nicola Bazzanella, Massimo Cazzanelli, Alessandro Barge, Michele Orlandi, Angelo Bifone, Antonio Miotello

**Affiliations:** 1 Department of Physics, University of Trento, via Sommarive 14, 38123 Povo, Italy; nicola.bazzanella@unitn.it (N.B.); massimo.cazzanelli@unitn.it (M.C.); michele.orlandi@unitn.it (M.O.); antonio.miotello@unitn.it (A.M.); 2 Center for Neuroscience and Cognitive Systems, Istituto Italiano di Tecnologia, corso Bettini 31, 38068 Rovereto, Italy; mirko.sacco@unito.it (M.S.); angelo.bifone@iit.it (A.B.); 3 Department of Drug Science and Technology, University of Torino, corso Raffaello 30, 10125 Torino, Italy; alessandro.barge@unito.it; 4 Department of Molecular Biotechnologies and Health Sciences, University of Torino, via Nizza 52, 10126 Torino, Italy

**Keywords:** nitrogen-vacancy center, nanodiamonds, laser ablation, optically detected magnetic resonance

## Abstract

Due to the large number of possible applications in quantum technology fields—especially regarding quantum sensing—of nitrogen-vacancy (NV) centers in nanodiamonds (NDs), research on a cheap, scalable and effective NDs synthesis technique has acquired an increasing interest. Standard production methods, such as detonation and grinding, require multistep post-synthesis processes and do not allow precise control in the size and fluorescence intensity of NDs. For this reason, a different approach consisting of pulsed laser ablation of carbon precursors has recently been proposed. In this work, we demonstrate the synthesis of NV-fluorescent NDs through pulsed laser ablation of an N-doped graphite target. The obtained NDs are fully characterized in the morphological and optical properties, in particular with optically detected magnetic resonance spectroscopy to unequivocally prove the NV origin of the NDs photoluminescence. Moreover, to compare the different fluorescent NDs laser-ablation-based synthesis techniques recently developed, we report an analysis of the effect of the medium in which laser ablation of graphite is performed. Along with it, thermodynamic aspects of the physical processes occurring during laser irradiation are analyzed. Finally, we show that the use of properly N-doped graphite as a target for laser ablation can lead to precise control in the number of NV centers in the produced NDs.

## 1. Introduction

Nitrogen-vacancy (NV) centers attracted great interest and research activity for many emerging applications in quantum technologies [1,2,3] due to the unique properties of this color center, such as its extremely long spin-coherence times [4] and easiness in spin manipulation and readout [5]. For this reason, proposed applications are in imaging [6,7] and in the use of NV as a qubit for quantum information protocols [8]. Furthermore, of great promise is the employment of NV for quantum sensing applications [9,10,11], thanks to NV fluorescence dependence on environmental parameters [12]. Excellent mechanical and optical properties [13], as well as biocompatibility [14], allowed nanodiamonds (NDs) to become a relevant framework for hosting NV centers. In particular, regarding quantum sensing, NV-hosting NDs allow the detection of environmental parameters with nanoscale precision and sensing of magnetic fields [15,16], temperature [17,18], presence of paramagnetic species [19], changes in pH or redox potential [20] have been demonstrated. Moreover, the interaction between NV centers’ electronic spin and crystal strain of the diamond host allowed the realization of hybrid quantum systems based on NV centers coupled to a mechanical resonator [21], as well as spin-based strain imaging devices [22]. For the development of these applications, the production of NDs remains a challenge, given the difficulties and drawbacks of the standard production techniques, namely detonation [23] and milling [24]. These procedures do not allow good control in tailoring NDs size [25] and nitrogen doping concentration [26]. Moreover, postsynthesis processes are needed [27] to clean the NDs from impurities—as usually strong chemical treatments are required [28,29]—and nitrogen implantation is necessary to increase the number of NV centers in the nanocrystals [30]. Recently, an interesting prospect to control nitrogen doping during NDs synthesis was proposed, consisting of chemical vapor deposition (CVD) at a moderate level of N_2_ [31]. Furthermore, other top-down fabrication methods were demonstrated, based on a combination of CVD and plasma etching, leading to the formation of single-crystal NDs with uniform size of 30.0 ± 5.4 nm [32] and nanoscale cylindrical (down to 100 nm diameter and 500 nm height) diamond particles [33]. Of great importance for sensing applications is the production of NDs with a highly dense NV ensemble to increase detection efficiency. As a matter of fact, sensitivity scales as N^−1/2^ with N being the number of NV centers [4]. The standard way to obtain highly NV-dense NDs requires a multistep post-synthesis treatment of the NDs, consisting of ion/electrons irradiation followed by annealing at high temperatures [30]. An alternative is represented by laser ablation, which was proven to successfully convert graphite into NDs with a limited size distribution (≤100 nm) [34,35]. Furthermore, ablation of a carbon precursor immersed in a nitrogen-containing medium brings to highly NV-fluorescent NDs, without the need for secondary treatments to improve the NV centers’ density [36,37]. In this paper, we compare the different NV centers’ production efficiency of the recently developed fluorescent NDs laser ablation synthesis techniques, namely the ablation of the N-doped graphite in water [35], gaseous [36] and liquid nitrogen (LN_2_) [37]. Firstly, the actual formation of NV containing NDs through laser ablation of the N-doped graphite target in water is proved. The produced sample is fully characterized in its structural and optical properties. In particular, it shows a strong photoluminescence (PL) emission and a typical NV^−^ center optically detected magnetic resonance (ODMR) fingerprint, thus proving the NV-hosting NDs formation upon laser ablation. The N-doped graphite target is then laser-ablated in different environments (namely N_2_ atmosphere and LN_2_), to study possible additional nitrogen content due to the medium effects on NDs fluorescence intensity (namely on NV density). The different observations are reported, showing that ablation in LN_2_ brings the best NV-centers-enriched NDs production efficiency. Furthermore, the main aspects of the thermodynamic processes explaining this result are illustrated. Finally, we showed that this particular N-doped target could open a way to obtain NDs with a tunable concentration of NV centers.

## 2. Materials and Methods

### 2.1. N-Doped Graphite Target Preparation

Graphite powder (median size 7–10 μm) was purchased from Alfa Aesar (Ward Hill, MA, USA). Chemicals have been purchased in reagent grade from Sigma Aldrich (Saint Louis, MO, USA) and Alfa Aesar and have been used without further purification. The N-doped graphite powder was obtained performing the well-known 1,3-dipolar cycloaddition [38,39] via amino acid in situ-generated azomethine ylide (glycine or histidine) on the sp^2^ carbon graphite surface. The cycloaddition adduct, a pyrrolidine ring, brings the nitrogen atom required for graphite doping. More in detail: 1.102 g of graphite were suspended in a solution made by 0.550 g of paraformaldehyde (18.3 mmol, 1 eq) and 1.650 g of glycine (22.0 mmol, 1.2 eq) in 20 mL of DMF. The suspension was allowed to react for 2 h at 130 °C under magnetic stirring. After cooling to room temperature, the reaction mixture was centrifuged (4000 rpm, 8 min) and the supernatant was discarded. The graphite was subjected to several centrifugation cycles (water, 4000 rpm, 8 min, 3×; water/dioxane 1:1, 4000 rpm, 8 min, 3×; dioxane, 4000 rpm, 8 min, 3×). Drying at 70 °C for 24 h yielded the functionalized graphite, ready for thermogravimetric analysis. Another sample with a lower concentration of nitrogen was prepared, by changing the composition of the initial solution: 1.010 g of graphite were suspended in a solution of 2.52 g of p-nitrobenzaldehyde (16.7 mmol, 1 eq) and 3.080 g of histidine (20.0 mmol, 1.2 eq) in 45 mL of DMF. Finally, 200 mg of the N-doped graphite powder was pressed to 50 bar to form a target of 1 cm diameter and 1 mm thickness.

### 2.2. Pulsed Laser Ablation

Pulsed laser ablation of the N-doped graphite target is performed by using a KrF excimer laser (Lambda-Physik Coherent LPX220i) having a wavelength of λ = 248 nm, pulse duration τ = 20 ns and repetition rate of 10 Hz. Laser ablation was performed in different confining media: water, LN_2_ and nitrogen atmosphere. For the ablation in liquid phase (water and LN_2_), the target was placed in a glass vial, which was then filled with the liquid till a ~5 mm thick liquid layer covers the top surface of the target. Regarding ablation in LN_2_, to limit the unavoidable LN_2_ evaporation, the glass was in turn placed in a polystyrene box filled with LN_2_. The laser beam was focused on the target surface with a 40 cm-focal length lens, to form a laser spot size of ~1 mm^2^. Laser single-pulse energy was set to ~500 mJ, for a total of 3000 pulses. Once the ablation was performed, the target was removed from the vial, and the liquid-suspended powder was slowly dried in an oven. Finally, the powder was dispersed in 250 μL of isopropanol and then 2 μL were deposited on a substrate for characterization. For the N-doped graphite target ablation in nitrogen atmosphere, the target was mounted inside a vacuum chamber, which was brought to high vacuum conditions (residual pressure 10^−4^ Pa). Afterwards, the chamber was filled with N_2_ gas, which was fluxed through a fluxmeter to attain a partial pressure of 1 Pa. Laser ablation was performed with the same KrF laser, operated in the same configuration (laser energy, spot size, number of pulses) described above for ablation in liquid, in order to compare samples synthesized in the same nominal conditions. The ablated material was deposited onto a silicon substrate positioned in front of the target at a distance of 10 cm. The initial cleaning procedure of the silicon substrates included three subsequent baths in ultrapure acetone, ethanol and deionized water in an ultrasonic bath (10 min for each passage) and then dried with gaseous nitrogen flux. The silicon substrate was mounted on a heated holder and, during deposition, the temperature was kept at ~100 °C. After the deposition, the film was annealed at 300 °C for 1 h, in order to reduce the internal stresses of the film and avoid delamination.

### 2.3. Characterization Techniques

To measure nitrogen concentration in the target, thermogravimetric analysis (TGA) was performed, since it provides information about the thermal stability of surface groups. The derivatization amount has been calculated from weight loss percentage differences at 650 °C (where the adduct is completely removed), with respect to the untreated graphite, and expressed in terms of mol/g. Nitrogen doping amount has been expressed in terms of grams of nitrogen per gram of graphite, calculated from the molar amount of pyrrolidine adduct per gram of graphite. Thermogravimetric measurements were carried out on a TGA 4000 analyzer (Perkin Elmer, Waltham, MA, USA) under a high purity argon atmosphere, in the temperature range 50–800 °C, with a heating rate of 10 °C min^−1^ (ca. 10 mg of sample were used for each analysis). Morphological and compositional analysis of the laser-synthesized samples was performed with a JEOL LSM-7001F Field Emission Scanning Electron Microscopy (SEM), using a working distance (WD) of 10 mm and with an energy beam of 20 keV. Raman and photoluminescence characterizations were made with a Jobin-Yvon Horiba LabRam Aramis confocal micro-Raman system, operated in the following set: confocal hole 1000 μm, slit width 100 μm and grating 1800 lines/mm. The excitation source was a DPSS laser with 532 nm wavelength, and the signal was collected with an air-cooled multichannel CCD in the range 100–8000 cm^−1^ (corresponding to 535–926 nm with a 532 nm excitation), with an accuracy of ±1 cm^−1^. Wide-field PL imaging was performed with a Nikon Ti-E inverted wide-field microscope using a 532 nm laser pump (model: CNI laser mod. MGL-III-532/50 mW). The PL signal was collected by a 40× (NA = 0.75 and 0.66 mm working distance) refractive objective, and then filtered by a TRITC (Tetramethylrhodamine) dichroic beamsplitter, before being measured by an sCMOS camera (ORCA-Flash4.0 V2, Hamamatsu, Hamamatsu City, Japan). ODMR spectra are obtained with the same apparatus: under continuous laser excitation, the RF signal, which was obtained with a WindFreak RF generator (SynthHD v1.4 54 MHz–13.6 GHz) and amplified with a Mini-Circuits ZVE-3W-83+ 2W RF, are delivered on the sample with a homemade Au-coated copper loop terminated with a high power RF dumper. The temporal sequence of the experiment (RF delivery and camera acquisition) was obtained by suitably programming a SpinCore 100 MHz TTL generator (Mod. TTL: PB12-100-4K). Finally, the collected images are processed with the Nikon software NIS-Elements Advanced Research.

## 3. Results

### 3.1. NDs Synthesis and Characterization

**Figure 1:**
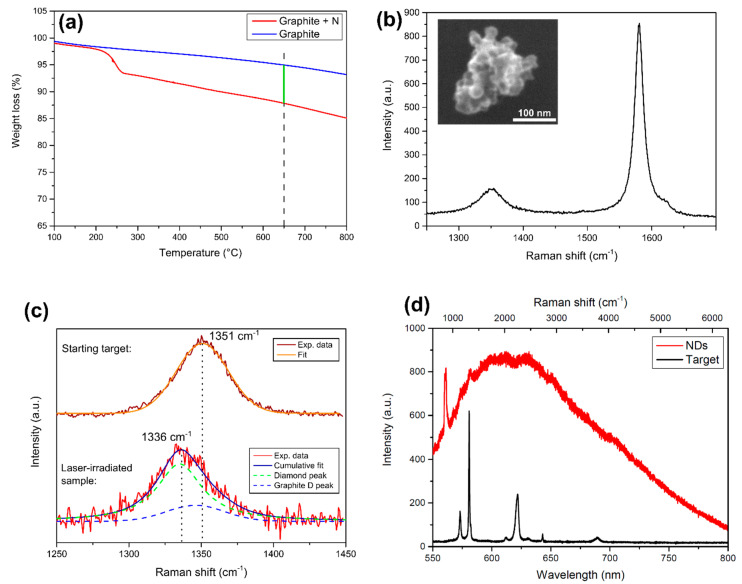
Characterization of the sample. (**a**) TGA analysis of the N-doped graphite target. The weight loss of the derivatized sample (red line) respect to the untreated graphite (blue line) is measured at the temperature of 650 °C (green line), to extract nitrogen content. (**b**) Raman spectrum of the ablated powder under 532 nm laser excitation, dominated by the so-called graphite G peak at ~1580 cm^−1^. The left peak is detailed in the next panel. Inset: morphological SEM analysis of the powder resulting from laser ablation in water: clustered nanoparticles having size of <100 nm are observed. (**c**) Effect of laser ablation on graphite D peak of the starting N-doped graphite target. Top spectrum: graphite D peak of the initial target, fitted by one gaussian (orange curve) centered at 1351 cm^−1^. Bottom spectrum: after laser irradiation, the peak shifts to lower wavenumber (1336 cm^−1^). It is composed of two Gaussians, one at 1335 cm^−1^ (green dashed line), attributed to compressively-strained nanodiamonds (NDs), and one at 1346 cm^−1^, which is the graphite D peak (blue dashed line). The convolution between these two (red line) well fits the experimental data (black line). (**d**) PL spectrum of the NDs (red curve) obtained under 532 nm laser pumping. The sharp peak at 561 nm (corresponding to 970 cm^−1^) is the second-order Raman peak of the silicon substrate where the powder is dispersed. The black curve represents the PL/Raman spectrum of the starting N-doped graphite target under the same laser pumping conditions: no PL emission is observed. The sharp peaks are the Raman peak (see top x-axis for the corresponding Raman shift in cm^−1^) of graphite, from left to right: the peak at 573 nm (1351 cm^−1^) is the graphite D peak, while the one at 580 nm (1580 cm^−1^) is the graphite G peak. The next triplet is formed by the central 621 nm (2704 cm ^−1^) 2D graphitic peak with the two side mirrors D+D’’ at 612 nm (2464 cm ^−1^) and D+D’ at 630 nm (2927 cm ^−1^). The other two are the 2D’ and 2D+G’ at, respectively, 643 nm (3241 cm ^−1^) and 689 nm (4279 cm ^−1^) [40].

The N-doped graphite powder, subsequently pressed to form the target for laser ablation, was studied with TGA to quantify nitrogen concentration achieved during derivatization. As can be seen in Figure 1a, a weight loss is observed at ~230 °C compared to untreated graphite, due to the removal of the pyrrolidine ring, thus proving successful derivatization. To quantify the nitrogen amount obtained in the sample, the percentage weight loss was calculated at 650 °C: from this value, and knowing the molar weight of the adduct used, a nitrogen concentration of 23.2 mg/g of graphite is reached in the derivatized sample. The pellet made with this N-doped powder was then used as a target for laser ablation in water. SEM morphological analysis of the ablated material is reported in the inset of Figure 1b, showing clustered nanoparticles having size <100 nm. Raman spectroscopy analysis is reported in Figure 1b, where it can be seen that the spectrum is still dominated by the graphite G peak at ~1580 cm^−1^. The other peak, at 1336 cm^−1^, is highlighted in Figure 1c bottom spectrum (red curve), in which it is proved the presence of NDs in the ablated material. Indeed, the peak at 1336 cm^−1^ is a convolution between the diamond peak (green dashed line) at 1335 cm^−1^, and the residual graphitic D peak (blue dashed line) at 1346 cm^−1^ [40], originating from the sp^2^ carbon atoms surrounding the NDs cluster. To further confirm the presence of diamond phase, Figure 1c provides a comparison with the graphite D peak of the starting target (brown curve). The peak is at higher wavenumber (1350 cm^−1^) and it is well fitted by a single gaussian, meaning that no diamond phase is present in the initial target.

The blue-shift of the NDs Raman peak respect to the 1332 cm^−1^ bulk diamond feature is related to residual compressive strain acting on diamond nanocrystals [41]. NDs peak broadening (FWHM ≈ 44 cm^−1^) was already observed in untreated detonation NDs showing Raman peak FWHM > 30 cm^−1^ [13], due to high sp^2^/sp^3^ [42] ratio in the NDs clusters and partial amorphization of the nucleated NDs [43]. Both these two observations are consistent with NDs embedded in a graphitic matrix, as in our case. Finally, both SEM analysis and Raman characterization are consistent with NDs obtained through the ablation of pyrolytic graphite in different liquid environments [35,37]. Figure 1d shows the PL spectrum, under continuous 532 nm laser excitation, of the starting N-doped graphite target (black curve) and the NDs (red curve). In the latter case, a broad band is observed, consistent with the already reported NV^−^ emission [44,45] shifted toward the low-wavelength region due to the presence of graphitic defects on the NDs surface [46]. Photoexcited electrons are trapped by the sp^2^ carbon atoms on NDs surface [47], thus leading to the alteration of the typical NV^−^ emission spectrum. This is a first proof that the nitrogen contained in the target is incorporated in the forming NDs upon laser irradiation. On the opposite, the starting target does not show any PL emission, as only the Raman peaks of graphite (as described in the experimental section PL and Raman analysis are performed with the same apparatus) are detected. The full interpretation of the Raman spectrum is provided in the caption of Figure 1.

**Figure 2:**
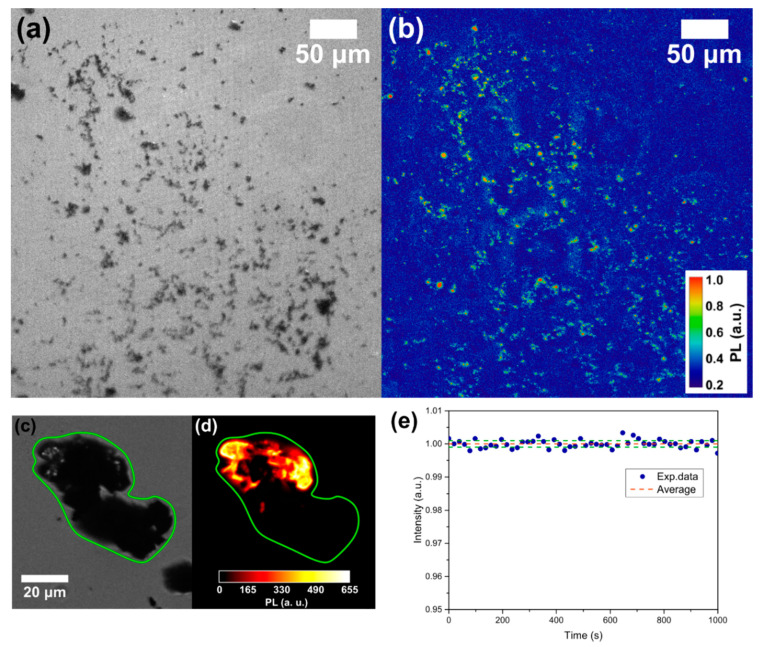
(**a**) Wide-field obtained with a white lamp and (**b**) PL imaging with a 532 nm excitation source of the same part of the sample. The bright spots in (**b**) are nitrogen-vacancy (NV)-fluorescent NDs. Detail of the same cluster in wide-field (**c**) and PL imaging (**d**). (**e**) PL stability measurement: normalized PL intensity (blue dots), average PL value (red line) and 2σ standard deviation interval (green line).

**Figure 3:**
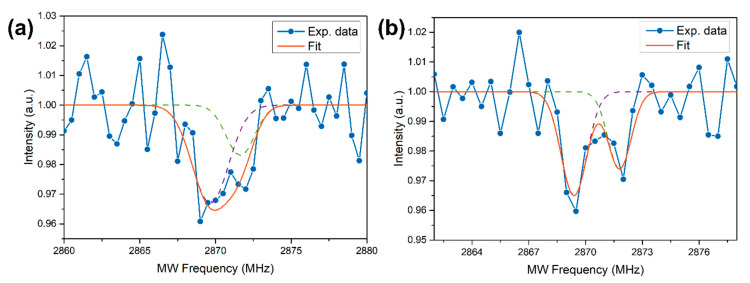
(**a**,**b**) Two typical optically detected magnetic resonance (ODMR) spectra, where the blue dots are the experimental data, while the orange line is the cumulative fit in turn made by the superposition of two Gaussians (dashed lines). The different shifts in the peaks composing the spectra are related to different strain fields acting on the NDs hosting the NV centers.

Wide-field imaging analysis is shown in Figure 2a for the bright field and in Figure 2b for the fluorescent imaging (532 nm excitation source) of the same sample region. The powder shows a strong PL, but a part of the ablated material does not present any emission. For example, an optical image of a micrometer-sized cluster is given in Figure 2c while the corresponding PL image of the same particle is reported in Figure 2d: as observed, only a portion of it presents a red emission. This means that, as expected, not all the ablated graphite is converted into NDs. More quantitatively, by comparing the PL images with the corresponding optical image, the synthesis efficiency of the fluorescent NDs/graphite was calculated. A rough estimate, given by the ratio between the area showing PL emission of Figure 2b and that covered by graphite powder in Figure 2a brought to the efficiency of fluorescent NDs synthesis of 6–7%. This result is consistent with the reported conversion efficiency of 5% reported by Yang et al. [48] for NDs from the ablation of graphite in water. To check if the PL emission is consistent with NV centers, and does not originate from other fluorescent sources such as carbon quantum dots, we performed a PL stability measurement. Indeed, it is well known that NVs present a long photostability compared to other carbon structures. The result is reported in Figure 2e showing the normalized PL intensity (blue dots) together with the 2σ standard deviation interval (green lines) of the data around the average value (red line) under continuous 532 nm laser excitation. Excellent temporal stability of the emission over a 10 min timescale can be observed, proving the quality and the origin of the NV fluorescence from our NDs. To finally prove NV center formation inside the NDs, ODMR spectroscopy is performed over the bright spots observed with PL imaging (Figure 2b). Figure 3 reports two typical ODMR spectra obtained by monitoring the PL intensity under continuous laser excitation while sweeping an MW field in the 2800–2900 MHz. Both spectra show a decrease in PL emission at around 2870 MHz, thus proving the NV^–^ origin of the observed PL [4]. The different split in the peaks composing the ODMR is a consequence of the different strain field acting on the NDs hosting the NV centers [36]. Furthermore, the strain effect is a consequence of NDs limited size, and zero-field splitting in the order of a few MHz was already observed for HPHT-NDs with a median size of 50 nm [49].

### 3.2. NDs Production Yield: Role of the Confining Medium

Now that the synthesis of NV-enriched NDs through pulsed laser ablation of the N-doped graphite is proved, we use this target to compare fluorescent NDs synthesis efficiency of different laser-assisted production techniques, that are: laser ablation in water, in LN_2_, and in nitrogen atmosphere. The comparison is performed by preparing three samples using the same N-doped graphite target which is laser irradiated in the three confining media. As described in the experimental section, the samples are produced with the same nominal condition, just changing the confining medium, to allow a proper comparison and study the effect of the latter. Morphological and structural characterization of the ablated material when the N-doped graphite is ablated in LN_2_ and nitrogen atmosphere is not given here, since it is already fully described in our recent papers [36,37] for the case of pyrolytic graphite target. We do not expect any relevant difference with the use of this particular target, as observed in this work for ablation in water.

**Figure 4:**
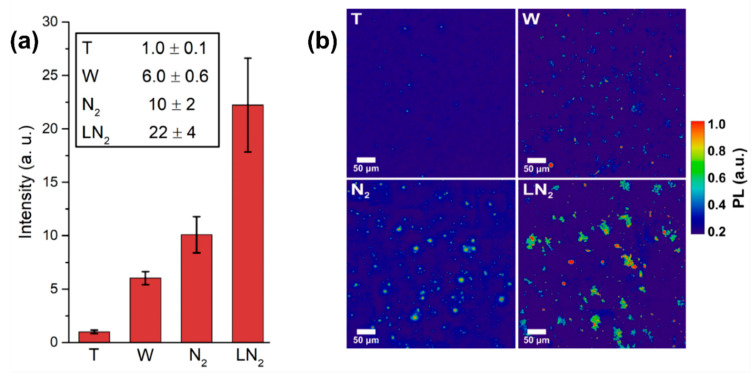
(**a**) NV-synthesis efficiency: comparison between different confining media. Different samples are labeled as: nonirradiated target (T), ablation in water (W), in nitrogen atmosphere (N_2_) and in LN_2_. For 10 images per sample, the fluorescence intensity is integrated after background removal. The resulting intensity is then normalized respect to the one of the initial target T. The error bars are the standard deviations of the pictures set. (**b**) Typical PL images of the samples after background subtraction.

To compare the different fluorescence intensities, PL imaging is used: from the PL pictures, such as the one shown in Figure 2b, the background is properly removed and then the total intensity of the image is integrated. For each sample, 10 images are collected and their integrated intensity is averaged. The result is reported in Figure 4a, where the PL intensity of the nonirradiated target (T), and the samples resulting from laser ablation in water (W), nitrogen atmosphere (N_2_) and LN_2_ are compared. Typical PL imaging pictures are reported in Figure 3b for the different samples. The best result in term of fluorescent NDs production efficiency is obtained for ablation in LN_2_, that shows an intensity 22 ± 4 times higher respect to the untreated target. Moreover, the LN_2_ sample shows a double intensity with respect to ablation in nitrogen atmosphere. This is a consequence of the different ablation plume expansion dynamics in a liquid or vapor medium [50,51]. The details of the thermodynamic model describing the formation of NDs in the two cases are deeply described in [36,37], here only a brief summary is given. When the ablation is performed in a dense environment such as a liquid, the thermodynamic conditions required for diamond phase formation are enhanced in the inner of the ablation plume [34,52]. These conditions are high pressure, high temperature and rapid quenching of the plume [53,54]. Regarding the former, the ablation process in LN_2_ brings the pressure inside the ablation plume to ≈3.5 GPa [37,55], much higher than the pressure of 220 MPa reached for ablation in a 1 Pa nitrogen atmosphere [36]. Since high pressures are required to form a diamond phase during laser ablation of graphite [56], a larger efficiency in synthesis of fluorescent NDs is expected for ablation in LN_2_ where cooling rate is of the order of 10^11–12^ K/s [57]. Whereas, regarding the quenching in the N_2_ atmosphere, the lifetime of the plume is much longer and collisional cooling is not very efficient compared to cooling in LN_2_. This longer lifetime leads to a larger sp^2^ carbon atoms contamination of the forming NDs, because chemical reactions in the plume occur when temperature and pressure are decreased and no longer the ones required for diamond phase formation. Graphitic layers covering NDs are reported to quench NV emission intensity [58], thus N_2_-atmosphere sample is expected to show weaker luminescence emission as observed indeed in Figure 4a. Lastly, the less luminescent sample is obtained for ablation in water. This can be easily explained considering that the fluorescence relies only on the nitrogen atoms contained in the target, which is subsequently incorporated by the NDs formed upon ablation in the water environment. On the other hand, ablation in a nitrogen-containing media brings to dissociation of N_2_ molecules of the liquid/atmosphere [36,37], providing an additional source of nitrogen doping, thus leading to a larger density of NV centers in the NDs.

### 3.3. Fluorescent NDs Production Yield: Role of the Target

**Figure 5:**
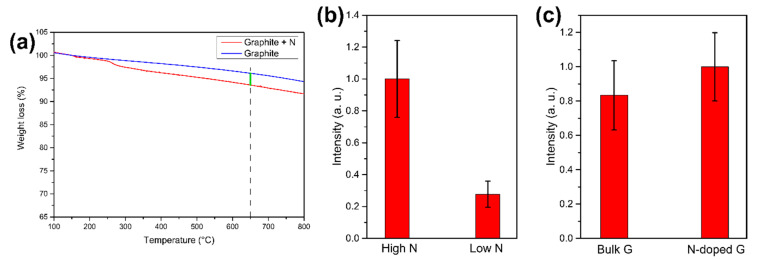
(**a**) TGA analysis of the target with low nitrogen doping (“Low N” target). Nitrogen content is extracted by measuring the weight loss of the N-doped graphite (red line) respect to untreated graphite (blue line) at 650 °C (green line). (**b**) Effect of different nitrogen doping in the target. PL intensities comparison between NDs laser-ablated in water from the “High N” and “Low N” target. (**c**) PL intensity comparison between laser ablation in LN_2_ of standard pyrolytic graphite and N-doped graphite target. In (**b**,**c**), the PL intensity is obtained by integrating 10 images after background subtraction. The error bars are the standard deviations.

To study the effect on NV-synthesis efficiency of nitrogen concentration in the target used for laser ablation, a graphite target with a lower N-doping level is prepared. With TGA analysis (Figure 5a) the nitrogen concentration is estimated to be 5.8 mg/g of graphite. This target, that will be referred to as “Low N”, is compared with the target described above, that from now on will be called “High N”. The latter, as described previously, has a nitrogen concentration of 23.3 mg/g of graphite, which is ~4 times higher respect to the “Low N”. To perform the comparison, the two targets are laser-ablated in water, and the obtained samples are analyzed with PL imaging. The ablation is performed in water because the effect of the initial nitrogen doping in the target is the aim of the experiment, so the laser irradiation is performed in a medium that does not bring to an additional nitrogen doping. The result of the PL imaging analysis is reported in Figure 5b: the “High N” sample shows a larger PL intensity, meaning that higher doping in the target does bring to a higher concentration of NV in the formed NDs. In particular, the ratio between the two samples PL intensity is 4 ± 1, similar to the ratio between the initial nitrogen doping. As a consequence, NDs fluorescence may be tuned by varying nitrogen doping in the target used for laser ablation: this result is the first step into establishing a route for the synthesis of NDs hosting a controlled number of NV centers. Finally, a last comparison is performed, with the aim of establishing if the dominant source of nitrogen atoms to form NV centers in the NDs is the nitrogen contained in the target or in the confining medium. To do that, two samples are prepared both by laser ablation in LN_2_: one by using as a target the nitrogen-doped graphite (“N-doped G” sample) the other by using standard pyrolytic graphite (“Bulk G” sample). The comparison, studied through PL imaging, is reported in Figure 5c: as can be seen, within the experimental error, the two samples have the same PL intensity. This means that, at the present doping level of the target, the most relevant source of nitrogen atoms to form NV centers in the NDs is LN_2_.

## 4. Conclusions

In conclusion, we have demonstrated the synthesis of NDs hosting NV^−^ centers through pulsed laser ablation of a nitrogen-doped graphite target. NDs are fully characterized in their morphological and crystal structure, as well as in the optical properties. Furthermore, the presence of NV centers in the NDs is unequivocally proved by optically detected magnetic resonance. Moreover, this particular target is used to compare different laser-ablation-based techniques recently developed by our group, namely laser ablation of graphite in LN_2_ and in a nitrogen atmosphere. The aim is to find the best condition for the production of NV-doped NDs, through the single-step, scalable and facile process provided by laser synthesis. The best result in terms of fluorescent NDs formation efficiency is obtained for ablation in LN_2_: an explanation of the physical processes leading to this outcome is provided.
